# Religiosity, stress, and depressive symptoms among nursing and medical students during the middle stage of the COVID-19 pandemic: A cross-sectional study in Morocco

**DOI:** 10.3389/fpsyt.2023.1123356

**Published:** 2023-02-23

**Authors:** Ismail Rammouz, Laila Lahlou, Zineb Salehddine, Omar Eloumary, Hicham Laaraj, Mina Ouhamou, Khalid Mouhadi, Jalal Doufik, Rachid Aalouane, Said Boujraf

**Affiliations:** ^1^Clinical Neurosciences Laboratory, Faculty of Medicine, Sidi Mohamed Ben Abdellah University, Fez, Morocco; ^2^Clinical Neuroscience, Innovation and Ethic (NICE) Laboratory REGNE, Medical School of Agadir, Ibn Zohr University, Agadir, Morocco; ^3^Departement of Psychiatry, Faculty of Medicine, Ibn Zohr University, Agadir, Morocco

**Keywords:** religiosity, spirituality, stress, depression, students

## Abstract

**Background:**

Recent studies on nursing and medical students showed a higher prevalence of depression and stress than the general population. Religiosity and spirituality are common in Muslim countries and are usually used as a means of coping strategy for psychological and mental disorders.

**Objective:**

Our objective was to evaluate the association between religious actions, depressive symptoms, and stress among students of nursing education lasting 3 years and students from the first 3 years of medical education lasting 7 years. The study was conducted at Ibn Zohr University of Agadir, Morocco.

**Method:**

A sample of different stages of nursing and medical students was recruited. Religiosity was assessed by Muslim Belief into Action (M.BIAC) scale. The depressive symptoms and stress were, respectively, assessed by the Beck Depression Inventory (BDI-II) and Perceived Stress Scale (PSS).

**Results:**

Four hundred and thirteen students participated in this study. Our results showed a high prevalence of depressive symptoms (62.2%) and stress (66.8%). The depression scores were higher in the following subsample categories: students in the first 2 years of studies, female medical students, and nursing students with significant differences. The recorded religiosity was greater among students without depression compared to students with depression (*p* < 0.001). In the multivariate regression, the BIAC score demonstrated religiosity as neither a risk factor nor a protective factor of depression.

**Conclusion:**

Religiosity constitutes a protective factor of depression and stress among nursing and medical students. This should improve the student's ability to cope with stressful situations during their training. Prospective studies are needed to further investigate this association and how religiosity improves mental health. This would contribute to improved academic performance and wellbeing among medical and nursing students.

## Introduction

Medical education is one of the most demanding training programs in both academic and emotional dimensions (1). Previous studies have reported higher rates of poor mental health among nursing students (1–6).

A recent meta-analysis reported a global prevalence of anxiety of 33.8% among medical students, which is significantly higher compared to the general population (1). An earlier review of 11 studies reported a prevalence of anxiety among medical students outside North American countries, ranging between 7.7 and 65.5% (4).

In addition, a systematic review by Rotenstein et al. reported an estimate of depression prevalence or depressive symptoms among medical students of 27.2%, while suicidal ideation represented 11.1% (3).

In addition, studies reported conflicting findings about whether depression and suicidality vary according to undergraduate year, sex, or other demographic and educational characteristics (2, 3).

Felicilda-Reynaldo et al. measured the quality of life and examined the predictive roles of religiosity and spirituality coping among nursing students from four countries. Their findings showed that frequent attendance to organized and non-organized religious activities leads to better physical and environmental domains; in addition, using non-organized religious activities frequently leads to improved psychological health. The same study concluded that more frequent use of religious coping strategies was associated with better physical, psychological, and environmental health, with improved social relationships (7).

The last two meta-analyses reported in 2022 obtained similar outcomes. Thus, the first meta-analysis done in China by Jin et al. included all cross-sectional studies on the prevalence of depression among Chinese medical students. The prevalence of depression among medical students in China was 27%. Sleep quality was an important heterogeneous source of depression. Medical students with sleep disorders were more likely to report depression more than three times compared with students without sleep disorders (8).

Additionally, the second meta-analysis was done by Li et al. (9). They analyzed a total of 64 studies, including 100,187 individuals. The common prevalence of depression and anxiety symptoms among college students was 33.6%. The highest prevalence of symptoms of depression was reported in Africa (40.1%), lower middle-income countries (42.5%), and medical students (39.4%). While the prevalence of anxiety symptoms was the highest in North America (48.3%), lower middle-income countries (54.2%), and among medical students (47.1%). The same meta-analysis found that the prevalence of depression and anxiety symptoms was higher in post-COVID-19 disease stages studies, especially after the global stage of lockdown (9). Another meta-analysis analyzed a total of 27 cross-sectional studies involving 8,918 nursing students and reported a high prevalence of depression of 34.0%. Significant differences in the prevalence of depression were noted for different age subgroups. The highest prevalence of depression among young students was 41.0%; the different geographic areas such as Asian nursing students demonstrated a higher prevalence of depression of 43.0% (10).

Many risk factors are reported and included the high competitiveness of medical school, simultaneous hospital training and lectures, overnight shift work, the large volume of knowledge, and the growth of medical, paramedical, logistical, and relational responsibilities (11–13).

Individuals have used a variety of coping strategies to deal with stressors, such as seeking support from social relationships, ruminating, venting, distraction, problem-solving, substance use, humor, and religion (14, 15).

Over the past two decades, studies focused on the potential impact of religion and spirituality in coping with stress. People often turn to prayer and other forms of religious or spiritual observance in sickness stages, death, and other types of adversity. They often report retrieving a sense of comfort in assets such as religious and spiritual resources (16–18). Indeed, worldwide studies have reported that various aspects of religiosity/spirituality are improving depression symptoms and decreasing the incidence of suicide (13). Studies conducted in Muslim countries focused on the context and why religiosity should be considered a valid coping strategy for mental health issues (19, 20). In the literature, the terms religiosity and spirituality are commonly used. Religiosity is usually considered to include three dimensions including organizational religious activities (Mosque attendance), non-organizational activities (private religious activities), and intrinsic or subjective religiousness, while spirituality often includes a sense of transcendence beyond one's immediate circumstances and meaning in life (21, 22).

To achieve the objectives of our study, we used a measurement of religiosity that emphasizes religious actions more than the concept of spirituality; hence, we often use the term religiosity in our manuscript. Sociologists typically measure religiosity using indicators of belief, behavior, and belonging. However, the socio-religious history of the Islamic world is complex, and a renewed examination of the dimensions of religiosity is necessary.

In Morocco, Islam is the dominant religion. It is practiced according to the Sunni tradition of Islam Religion. Nevertheless, spirituality coping has never been studied among Moroccan students. Our goal was to estimate the religiosity level, stress, and depression and to find the correlation between religiosity and reported psychological disorders among nursing and medical students in the very specific middle stage of the COVID-19 pandemic after the global lockdown. We hypothesized that religious actions might constitute an alternative coping strategy for affective disorders in the context of Arabic Islamic countries during the global COVID-19 pandemic context. A hypothesis was contrived by focusing on the indices of stress, depression, and religiosity, each following the appropriate and validated measuring scale in the same context. In addition, two negative hypotheses were considered in our study. First, there is no significant relationship between religiosity and the stress of students in the context of the COVID-19 pandemic, and second, there is no significant difference between the behavioral dimensions of religiosity and depression of students during the COVID-19 pandemic stage.

## Methods

### The study population

The data were collected from two schools of the Ibn Zohr University of Agadir, Morocco including a Medical School and a Nursing School. Data collection was conducted between February and May 2021, i.e., 1 year after the national global COVID-19 lockdown and 8 months after the restricted COVID-19 opening. Medical education lasts for 7 years and is typically divided into three stages: the first stage with the first 2 years is mainly of fundamental sciences, the second stage with 3 years is fully dedicated to clinical sciences and hospital training, and the third stage with 2 years is fully dedicated to an internship program. The nursing education pathway requires 3 years of both 50% of academic courses and 50% of supervised clinical practice in a hospital.

### Data collection and inclusion criteria

The level of depression variable error acceptable was 5% (d = 0.05). In addition, the expected proportion of the population was 0.27 (*P* = 0.27) (8). Type I error rate was 5% (α = 0.05), and the minimal sample size of the survey was 303 (23, 24). Furthermore, we have accomplished weighting of the number of nursing students and medical students. The inclusion criteria of students were living in Agadir city, being Muslim, born in Morocco, speaking the Arabic language, being older than 18 years, and being free of any significant mental, neurological, and cognitive disorders. The collected data covered a representative sample of the first 3 years of medical studies and all semesters of nursing studies including 3 years. Our sample participants were randomly recruited without targeting a sex ratio of 1:1. In fact, the rates of female students in nursing and medical schools are high, ranging from 65 to 70%.

### Data collection process

Data collection was done during class time, not before or after educational activities. The questionnaire, objectives of the study, and confidentiality of data were well-explained by the investigators. All students participating in the study were informed of the study details including the protection of anonymous personal data and then gave their written informed consent. Administrative approval was obtained from the president of Ibn Zohr University to perform this study within the department of medical and nursing schools.

Our questionnaire initially targeted sociodemographic aspects such as age, sex, year of study, socioeconomic level of parents, marital status of parents and place of life, repetition of the classes' levels, and sources of study finance.

### Religiosity assessment

Religiosity was assessed using Muslim BIAC (M.BIAC) scale (25). The original study of BIAC in English was conducted on female caregivers living in the Southeastern and Western USA. This study demonstrated that BIAC has solid psychometric properties with excellent internal consistency, test–retest reliability, and convergent validity (26).

The BIAC consists of 10 questions, with each rated on a scale ranging from 1 to 10, except the first question which is scored 1 or 10 depending on the response. The total scale score ranges from 10 to 100. Item 1 directly asked respondents to choose their highest priority in life, with common priorities among the response options ranging from their health to their family (including God). The remaining items assess attendance at religious services, religious social involvement besides attending religious services, decision to place life under God's requirements, percentage of annual income given two religious causes, time spent listening/viewing religious media, time spent reading religious books and scriptures, time spent in prayer or meditation, time spent in religious volunteering, and the degree to which life is being conformed to one's religious teachings (27). The Arabic version of BIAC was published in 2016 (28).

Rammouz et al. (25) studied the Moroccan Arabic version of the Muslim BIAC on a sample of 132 students at Ibn Zohr University, Agadir, Morocco. The Cronbach's alpha for internal reliability was 0.81, with the alpha for removed items ranging from 0.77 to 0.82. Test–retest reliability by intra-class correlation coefficient (ICC) was 0.87 (95% CI = 0.83–0.91). Discriminant validity indicated relatively weak correlations between depressive symptoms (*r* = −0.06) and perceived stress (*r* = 0.08) (25).

#### Depression assessments

Depression was assessed using the Beck Depression Inventory short version (BDI-II) (29). The short form of the Beck Depression Inventory (BDI-13) is useful for screening and assessing depression in clinical and research conditions. The BDI-13 assesses the symptoms including depressed mood, pessimism, sense of failure, lack of satisfaction, self-guilt, self-hate, self-harm, social withdrawal, distorted body image, indecisiveness, work difficulty, fatigue, and loss of appetite. Abdel-Khalek translated BDI-II and studied the coefficient of alpha among samples of male and female undergraduates recruited from Egypt, Saudi Arabia, Kuwait, and Lebanon (*n* = 100, 80, 100, 100, respectively). Values of Cronbach's alpha were 0.77, 0.82, 0.89, and 0.67, respectively (30). The total score varies from 0 to 39. We considered the interpretation of the scores as follows: 0–3: no depression, 4–7: mild depression or light depression, 8–15: moderate depression, and 16 and above: severe depression. We recorded the BDI-II score into a dichotomous variable: without depression for the categories no and mild depression and the presence of depression for the categories moderate and severe depression.

### Stress assessments

The Perceived Stress Scale (PSS) was used in the sample (31). The PSS consists of 10 items and allows for assessing the stress perceptions over the past month for each participant. Each item is scored on 5 key choices of the Likert scale (0 = never to 4 = very often). A higher total score indicates higher levels of perceived stress. The translation and validation into the Moroccan Arabic dialect of PSS were completed by Ben Loubir et al. on 535 participants aged over 18 years and belonging to different social categories. The Moroccan version of the PSS showed good internal reliability (Cronbach's alpha = 0.80) and test–retest reliability (ICC = 0.87) (32).

### Data analyses

The statistical analysis was performed using Jamovi (open statistical software for the desktop and cloud software). All statistical methods used were two-tailed, with an alpha level of 0.05. Descriptive analysis was conducted by number (population) and percentage for categorical variables including students' sociodemographic data, perceived stress, and BDI-II severity. Cross-tabulation for factors associated with depression symptoms among students was determined using Pearson's chi-square test for significance and Fischer's exact test for small frequency distributions. Results are reported in Table 1. The mean of BIAC scores was compared with a *t*-test for 2 independent samples or ANOVA. For more than 2, a Tukey's test was carried out using *post-hoc* analysis. Prevalence and confidence intervals were estimated by the esci jamovi module. A multiple logistic regression model was used to determine the factors associated with depressive symptoms. The adjusted calculated odds ratio was statistically significant, with a *p*-value ≤ 0.05. All variables included in the multivariate analysis were statistically significant in the univariate analysis. They have also forced the model to introduce the BIAC score.

**Table 1:** Comparison of depression with sociodemographic data, religiosity, depression, and stress scores (BIAC).

**Variables**	**Total (*n* = 156413)**	**With depression *n* = 156257**	**Without depression *n* = 156**	***P*-value**
Age (years)	20.3 ± 1.68	20.2 ± 1.3	20.4 ± 2.1	0.08
Sex				<0.001
Female	289 (70)	196 (76.3)	93 (59.6)	
Male	124 (26)	61 (23.7)	63 (40.4)	
Repeated class	34 (8.3)	18 (8)	16 (10.4)	0.2
Students				<0.001
Medical students	92 (22.3)	38 (14.8)	54 (34.6)	
Nursing students	321 (77.7)	219 (85.2)	102 (65.4)	
Living				0.173
Alone	31 (7.5)	16 (6.2)	15 (9.6)	
With family	255 (61.7)	167 (65)	88 (56.4)	
With parents	121 (29.3)	69 (26.8)	52 (33.3)	
Campus	6 (1.5)	5 (1.9)	1 (0.6)	
Year of study				<0.001
First	102 (25.2)	79 (27)	23 (14.8)	
Second	174 (42.5)	112 (43.6)	62 (40.0)	
Third	133 (32.5)	64 (25.0)	69 (44.5)	
Marital statues				0.6
Single	403 (97.6)	251 (97.7)	152 (97.4)	
Married	7 (1.7)	5 (1.9)	2 (1.3)	
Divorced	3 (0.7)	1 (0.4)	2 (1.3)	
Parents situation				0.9
Married	366 (88.6)	227 (88.3)	139 (89.1)	
Divorced	17 (4.1)	11 (4.3)	6 (3.8)	
One dead	30 (7.3)	19 (7.4)	11 (7.1)	
Financing studies				0.01
Totally	244 (59.1)	166 (64.6)	78 (50)	
Partially	144 (34.9)	79 (30.7)	65 (41.7)	
No thing	25 (6.1)	12 (4.7)	13 (4.7)	
Socio-Economic				0.139
High	61 (14.9)	44 (17.1)	17 (11.1)	
Medium	288 (70.2)	172 (66.9)	116 (75.8)	
Low	61 (14.9)	41 (18)	20 (13.1)	
M.BIAC score	43.8 ± 14	43.6 ± 12	44.2 ± 16.6	0.6
Perceived stress				<0.001
Low	137 (33.2)	54 (24)	83 (53.2)	
Moderate	205 (49.6)	158 (61.5)	47 (30.1)	
Severe	71 (17.2)	45 (17.5)	26 (16.7)	

## Results

### Sociodemographic results

The achieved sample of the study consisted of 452 participants including 39 participants that did not complete the questionnaires. Thus, the final data to be analyzed consisted of 413 questionnaires collected from Muslims during the COVID-19 pandemic and represented a 28% higher sample compared to the initially calculated minimal representative sample. The mean age was 20.3 ± 1.68 years. The sample studied was predominantly women (70%), and 70.2% had medium family income. The details of the sociodemographic data are reported in Table 1.

### Depression and stress assessment results

The depression assessment reported 26.4% of respondents with scores of moderate depression and 35.8% reported severe depression. In addition, 49.6% of students reported moderate stress and 17.2% reported severe stress. Details of these assessments are presented in Table 2. The mean score of M.BIAC was 43.8 ± 14, and the median was 44, ranging between 35 and 52. More details are provided according to school affiliation, severity, and significance in Table 2.

**Table 2:** Levels of depression, stress, and religiosity among the study sample (BIAC).

**Variables**	**Total (*n* = 413)**	**Prevalence (CI95%) for nursing students**	**Prevalence (CI95%) for medical students**	**Nursing students (*n* = 321)**	**Medical students (*n* = 91)**	***P*-value**
M.BIAC score	43.8 ± 14* 44 [35–52]^∧^			43.1 ± 11.8	46.8 ± 19.3	0.025
Perceived stress score						<0.001
Low	137 (33.2)	38 (32.8–43.4)	16.5 (10.3–25.4)	122 (9)	15 (16.5)	
Moderate	205 (49.6)	53.9 (48.4–59.3)	35.2 (26.1–45.5)	173 (53.9)	32 (35.2)	
Severe	71 (17.2)	8.1 (5.5–11.6)	48.4 (38.4–58.5)	26 (8.1)	44 (48.4)	
Beck depression score						<0.001
No depression	97 (23.5)	21.2 (19–22, 24, 25, 28–31)	30.8 (22.2–40.8)	68 (21.2)	28 (30.8)	
Mild depression	59 (14.3)	10.6 (76.8–14.4)	27.5 (19.3–37.4)	34 (10.6)	25 (27.5)	
Moderate depression	109 (26.4)	23.1 (18.7–28)	38.5 (29.1–48.7)	74 (23.1)	35 (38.5)	
Severe depression	148 (35.8)	45.2 (39.8–50.6)	3.3 (1.1–9.2)	145 (45.2)	3 (3.3)	

^*^Mean ± SD. ^∧^Median [IQR]. CI, confidence interval.

Depression scores were higher in girls and nursing students, and in the first 2 years of studies at both nursing and medical schools, respectively with *p*-value < 0.05. Religiosity was shown to be higher in students without depression compared to students expressing depression (*p* < 0.001). Initial correlation analysis was done to characterize the relationship between depression and sociodemographic data. The detailed results are presented in Table 3.

**Table 3:** Binary multivariate logistic regression of factors associated with depression symptoms among medical and nursing students at Ibn Zohr University, Morocco, 2022.

	**ORa**	**CI (95%)**	***P*-value**
Nursing-medical student	6.52	3.15–13.50	<0.001^*^
Female	1.55	0.90–2.63	0.054
Year of study			
First-third	4.13	2.13–7.99	<0.001^*^
Second-third	3.01	1.68–5.39	<0.001^*^
Perceived stress			
Moderate-low	6.47	3.78–11.07	<0.001^*^
Severe-low	8.08	3.47–18.81	<0.001^*^
Financing studies			
Partially-totally	0.70	0.43–1.15	0.1
Nothing-totally	0.61	0.21–1.77	0.3
BIAC score	0.99	0.98–1.01	0.92

* Means statistically significant values.

**Figure 1:**
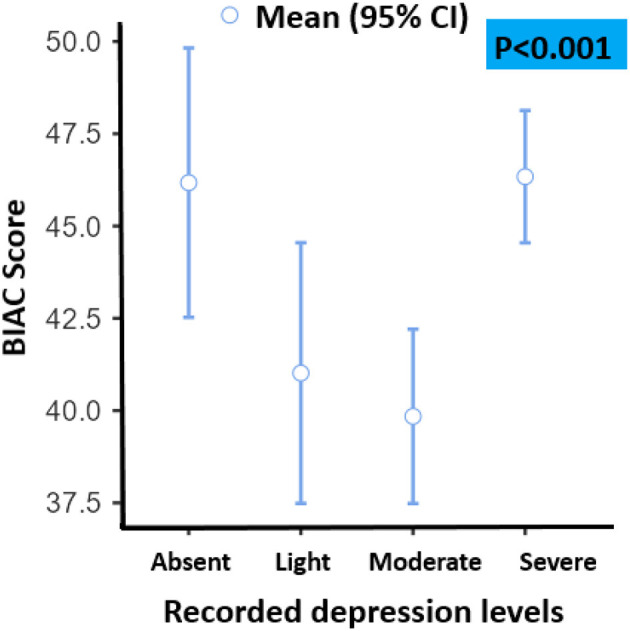
BIAC scores vs. depression levels. CI, confidence interval.

Indeed, the most achieved correlations of studied variables were found to be not statistically significant; except for depression which was found to be highly correlated with studying a nursing program vs. a medical program (*p* < 0.001). In addition, studying in the 2nd year was highly correlated with depression (*p* < 0.001). Details of these aspects are presented in Table 3. The difference between mean depression and mean stress was significant (*p* < 0.001). The religious scores were positively correlated with stress and depression scores. Therefore, we noticed an important finding; the religious scores were higher in students with either severe depression or without depression. In contrast, students with moderate depression have demonstrated the lowest religiosity scores (Figure 1). Moreover, the association between stress and religiosity did not reveal any significant difference between different levels of stress scores and religiosity scores (Figure 1).

**Figure 2:**
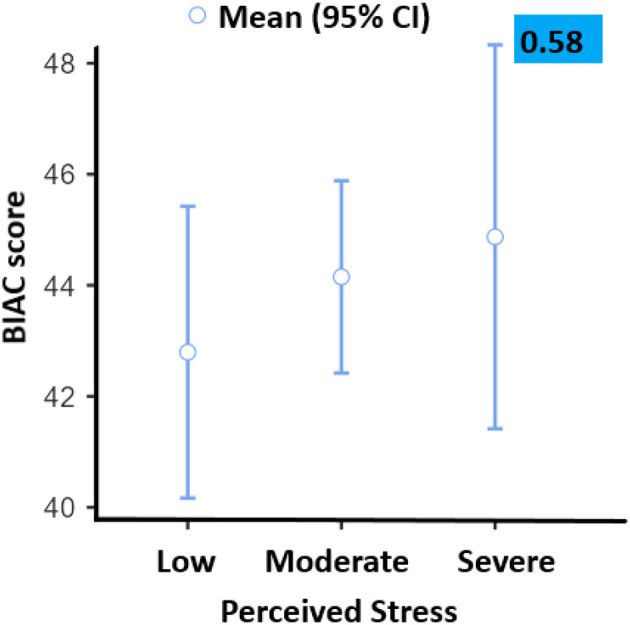
BIAC scores vs. stress levels. CI, confidence interval.

A binary multivariate logistic regression was done to examine the risk factors of depression symptoms among nursing and medical students (Table 3) found that nursing students have almost six times more risk for depression symptoms compared with medical students (OR = 6.52; 95% CI: 3.15–13.5) and students in the first (OR = 4.12; 95% CI: 2.13–8) or second year of study (OR = 3.01; 95% CI: 1.68–5.39) compared with the third year. In addition, the levels of depression and religiosity revealed in the study sample vs. severe (OR = 8.08; 95% CI: 3.47–18.8) and moderate (OR = 6.47; 95% CI: 3.78–11.07) perceived stress during the COVID-19 pandemic (Figure 2).

## Discussion

In fact, religiosity is probably used as a resilience factor by studied students with severe depression to fight against negative emotions and negative cognitions during the COVID-19 pandemic. Consistently, religiosity was higher among students without depression, indicating the potential use of religiosity as a defensive means against affective disorder during the COVID-19 pandemic. More explicitly,

i) Students with severe depression have expressed higher religiosity during the COVID-19 pandemic, and people with intense negative emotions are mostly indicating the style of religious coping.

ii) Students without depression during the COVID-19 pandemic have expressed higher religiosity, which could be explained by the preventive role of religiosity.

iii) Students with moderate depression during the COVID-19 pandemic have shown less religiosity; which could be explained by the fact that despite the presence of moderate negative emotions, these people do not feel a great need to have rescue remedy resources in religion.

BIAC is a tool for measuring religious behavior rather than any emotional or cognitive aspect of religion. Thus, our study has examined the religious actions with Islamic traditions during the COVID-19 pandemic. Indeed, religious coping is highly variable from one item to another. In Muslim countries, some people use their religious beliefs mostly within a cognitive framework, while others are involved in religious actions compared to general spirituality. These dimensions are supporting the concept of behavioral religiosity in opposition to emotional or/and cognitive religiosity, which is equivalent to the concept of spirituality. Reported studies have shown the link between spirituality/religiosity and affective disorders. The religiosity was associated with lower levels of anxiety, depressive symptoms, and demand for mental health services among black men on a college Campus (6) outside the COVID-19 pandemic context, while in our study, religiosity was shown to be significantly higher among students without depression and among students with severe depression.

Other studies have analyzed the potential protective factor of religiosity from suicidal behaviors (33, 34). In Tunisia, a Muslim country similar to Morocco, Romdhane et al. have studied Tunisian Muslim youth religiosity after the 2010 Revolution and found a strong negative correlation between suicidal ideations and the three sub-scores of religiosity after controlling for the associations between psychosocial variables and suicidal ideations scores (33). Jin Kim et al. studied college students who did not engage in religious meetings and private religious activities and showed to have elevated depressive symptoms and a higher risk of suicidal ideation (34). In addition, students are shown to have more confidence in an immense God rather than adhering to a given hard line of spirituality (35).

The relationship between religiosity and mental health is complex, and other intermediate factors may be crucial, such as personality features, resilience capabilities, social and family support, self-esteem, and many other potential factors.

Indeed, the different dimensions of religiosity are not equal regarding their perception and positive impact on mental health. Hence, the emotional dimension of religiosity approaching spirituality would be more crucial than the behavioral dimension.

Kendler et al. considered religiosity as a complex, multidimensional construct with substantial association with mental health. Some dimensions of religiosity are related to reducing the risks of mental disorders, especially impacting the potential of internalizing disorders, and others contribute to reduced risk, specifically in the sense of externalizing disorders. Kendler et al. (36) identified seven religious factors that impact mental health and the quality of psychological health, including general religiosity, social religiosity, involved God, forgiveness, God as judge, unvengefulness, and thankfulness.

In fact, the aspects of religiosity consist of behavioral dimensions, such as religious worship attendance and charity for religious matters; cognitive dimensions, such as thinking God has a big value in the person's life or thinking God as a judge; and emotional dimensions, such as meditation and feeling of fear of God, while the general spirituality is a concept integrating all these dimensions. In addition, the religious coping pattern is not always present, and it is used in different modes from one person to another. The coping mechanism depends on varying personality backgrounds, characteristics, and attitudes (35). Sultan et al. studied the effects of personality features on moderating the relationship between religiosity and the mental health of university students. They demonstrated that openness to experience and agreeableness as traits of students' personalities are significantly moderating the relationship between religiosity and mental health (37). In contrast, religious identity, self-esteem, positivity, and the presence of meaning in life are all involved in this complex relationship (38). Sakellari et al. performed a correlation analysis among the students of Cypriot University and found that greater levels of self-esteem were associated with lower depression levels and that stronger religious and spiritual beliefs correlated negatively with depression (39). Furthermore, Sakellari et al. investigated the correlation between health behaviors and dispositional optimism among nursing students in Poland, Spain, and Slovakia during the COVID-19 pandemic. This study showed that characteristic optimism is an important predictor of students' health behaviors (40). Further studies have reported that extra-curricular activities such as membership in hobbies clubs, cultural clubs, and sports clubs were shown to play a key role in developing interest and reducing stress in a student's life (35, 36). Belvederi Murri et al. (41) summarized a narrative review of relevant literature to address the aforementioned misperceptions and to provide practical recommendations for prescribing exercise to individuals with major depression. Indeed, a common misperception is that exercise is beneficial for depression mostly because of its positive effects on the body (“from the neck down”), whereas its effectiveness in treating core features of depression (“from the neck up”) is underappreciated.

Other factors might be also involved in the affective disorders among students, such as lifestyle. A study among nursing students in Ontario (Canada) showed that increased sitting time, poor sleep quality, and low dairy consumption were associated with higher scores of depression, anxiety, and psychological stress (13).

In our study, the depression scores were higher in the first 2 years of studies among both female medical students and nursing students with significant differences. Religiosity was greater among students without depression than those with depression, while the association between stress and religiosity showed no significant difference. Our results and reported literature suggested setting up a formal mental health prevention strategy for students in general and medical and nursing students in particular. In addition, stigma is also a major concern of such prevention settings consideration, treatment, and recovery from mental illness. Therefore, prospective studies must be carried out to analyze all measures in order to assess the efficiency of interventions and reduce stigma. Knowledge of the lines of research was carried out in various research institutions. The synergy between the different researchers and further multicenter studies within the framework of consortia would play an important role in reducing the stigma and improving care provided to nursing students with psychiatric diagnoses, considering the inclusion aspects (42). Sakellari et al. investigated the barriers to mental healthcare among nursing, pharmacy, and medical trainees in Nigeria and concluded that the co-existence of spiritual beliefs and biomedical and psychological models of mental health is a key factor that would allow reducing any effective stigma (12).

Finally, an integrative review has explored the vision and role in addressing students' mental health in nursing school students. Indeed, nurses do esteem their role as trusted members of the medical school community. The students perceived highly important their practice standards as an integral part of their position and recognized competence in mental health care. Future nurses, doctors, and healthcare students are not protected against mental disorders. Hence, helping students in an emergency, especially during the COVID-19 pandemic, has added supplementary stress to the already stressful context of education and work. Hence, different tools that allow confronting the challenges of the medical curriculum are obvious in general and in the COVID-19 pandemic context, and equivalent contexts. In addition, it is essential to facilitate asking help for students, which is often not possible due to factors such as the stigma of mental illness, confidentiality of the medical file, cost of psychiatric care, and lack of time (43). It is recommended to set up monitoring facilities and education resources throughout the nursing education period and introduce preventive practices for students' mental health (11). Practice recommendations should include providing the nursing school with evidence-based training on managing the mental health needs of students, as well as supporting access to nursing schools that can provide mental health supervision within the community (44).

The limitations of this study consist of a small study sample and less effectiveness of medical students and nursing students (medical school enrolled 459 students, whereas nursing school included 713 students). In addition, the rate of medical students was lower compared to the investigated rate of nursing students, with the medical students' recruitment limited to the first 3 years of the medical program, ensuring better matching to the nursing students dealing with an educational program of 3 years.

In addition, the female student population is over-represented, and as a result, there was an over-representation of women in the sample recruited; this is because the rate of female students in nursing and medical schools is high, ranging from 65 to 70%.

This final limitation is that all anxiety and depressive manifestations occurring within the last years of the medical student program population have not been considered.

Future studies should be carried out after the COVID-19 pandemic relapse to find the isolated effect of COVID-19 on stress and depression in medical and nursing students. This would allow to cover the need to find the effect of the medical and nursing environments on the psychological profile and religiosity in the Muslim community, especially those not yet enough studied.

## Conclusion

Religiosity constitutes a protective factor of depression and stress among nursing and medical students. Further studies of the effectiveness of religiosity in improving mental health, particularly among youth and university students, have to be supported. This will improve the student's ability to cope with stressful situations during their training. Otherwise, the stress and depression levels of nursing and medical students should be monitored and support should be oriented to help reduce these risk factors, in order to find the right place and the right people to treat their psychological suffering. More attention should be granted to religiosity coping with the prospective integration of psychological interventions by mental healthcare providers. Why not integrate religiosity coping in the future guidelines of the treatment of affective disorders? We suggest including in future nursing and medical schools' educational reforms a systematic and continuous screening of psychological/psychiatric disorders impairing integrating health studies among students.

## Data availability statement

The original contributions presented in the study are included in the article/supplementary material, further inquiries can be directed to the corresponding author.

## Ethics statement

The studies involving human participants were reviewed and approved by Fez Ethical commitee. The patients/participants provided their written informed consent to participate in this study.

## Author contributions

IR, JD, RA, and SB: concept and design and review. LL, ZS, OE, HL, MO, and KM: data collection, data analysis, and literature review. All authors contributed to the article and approved the submitted version.
